# Social Vulnerability and Traumatic Dental Injury among Brazilian Schoolchildren: A Population-Based Study

**DOI:** 10.3390/ijerph9124278

**Published:** 2012-11-22

**Authors:** Cristiane B. Bendo, Miriam P. Vale, Lícian D. Figueiredo, Isabela A. Pordeus, Saul M. Paiva

**Affiliations:** Department of Pediatric Dentistry and Orthodontics, Faculty of Dentistry, Federal University of Minas Gerais, Av. Antônio Carlos 6627, Belo Horizonte, MG 31270-901, Brazil; Email: crysbendo@yahoo.com.br (C.B.B.); miriamodonto@gmail.com (M.P.V.); lician_s48@hotmail.com (L.D.F.); isabelapordeus@ufmg.br (I.A.P.)

**Keywords:** tooth injuries, oral health, socioeconomic factors, environmental health, adolescent

## Abstract

The aim of the present study was to test the association between social vulnerability and the prevalence of traumatic dental injury (TDI). A population-based cross-sectional study was carried out with 1,556 schoolchildren aged 11 to 14 years in the city of Belo Horizonte, Brazil. The participants were examined for TDI using Andreasen’s criteria and those diagnosed with TDI were interviewed to determine the history of the injury. The Social Vulnerability Index (SVI) was used for socioeconomic classification, which addresses environmental, cultural, economic, legal and security/survival dimensions. The Poisson regression model was used for the multivariate analysis, with the significance level set at 5%. The prevalence of TDI was 14.1%; 59.3% of the participants with TDI did not seek a dentist after the incident. Poorer environmental, economic and legal conditions were statistically associated with the occurrence of untreated TDI (*p* < 0.05) and all the five SVI dimensions were associated with seeking a dentist due to TDI (*p* < 0.006). The prevalence of untreated TDI was higher among boys (PR: 1.42; 95%CI: 1.11–1.81) and those in situations of greater social vulnerability (PR: 2.27; 95%CI: 1.11–4.61). In conclusion, the male gender and high social vulnerability proved to be associated with the occurrence of TDI.

## 1. Introduction

The importance of socioeconomic conditions and one’s living environment has been studied over the few past decades in relation to the development of oral health problems [1,2,3,4]. The risk of harm to a child’s health may be increased due to environmental conditions, such as hazardous conditions of the home, neighborhood, playground, walkways, streets and school [1,4,5].

The impact of socioeconomic status on the occurrence of traumatic dental injury (TDI) is controversial in the literature [6]. The majority of studies addressing TDI have used family income, parents’ level of education, employment status and other socioeconomic indices to evaluate such an association [7,8,9,10,11,12,13]. Some studies have also demonstrated the importance of the neighborhood, environment and social capital with regard to the occurrence of TDI [1,2,3,4,14]. In conjunction with individual characteristics traditionally associated with TDI (boys suffer with more tooth injuries than girls and children with increased overjet have a greater prevalence rate of this condition) [7,9,13,15,16,17], the infrastructure of the area in which children live may also influence the prevalence of TDI [2,3,14]. Moreover, a number of studies have demonstrated a strong influence of the environment and social capital over the prevalence of TDI [2,3]. It has been found that boys living in areas with a high level of social capital have a lesser prevalence of TDI than those living in areas with a low level of social capital [2,3]. However, the same has not been found for girls [3]. 

Environmental hazards may explain differences in health conditions and such information can help guide health policies [5]. It is therefore fundamental to understand the influence of neighborhood infrastructure and families’ income and access to work, sanitation services, healthcare and education on the occurrence of TDI in children and adolescents. Local indices that consider these and other socioeconomic variables for the classification of living conditions are fundamental. As each country, region and city has its own peculiarities, such indices contribute to the understanding of associations between socioeconomic status and health. 

Given the controversial findings regarding the influence of socioeconomic status over the prevalence of TDI and the lack of studies on the permanent dentition using a detailed local index, the aim of the present study was to test the association between social vulnerability and the prevalence of TDI among Brazilian schoolchildren. The hypothesis was that TDI is more prevalent among schoolchildren from areas of greater social vulnerability.

## 2. Methods

### 2.1. Study Area and Design

This study received approval from the Human Research Ethics Committee of the *Universidade Federal de Minas Gerais* (Belo Horizonte, MG, Brazil). A letter was sent to the parents of the selected schoolchildren, explaining the aim, characteristics, importance and methods of the study and requesting their child’s participation. All parents and children who participated in this study signed a statement of informed consent. 

A representative cross-sectional study involving schoolchildren aged 11 to 14 years in the city of Belo Horizonte (southeastern Brazil) was carried out between August 2008 and April 2009. Belo Horizonte is the capital of the state of Minas Gerais. It is the sixth largest city in the country, with 2,375,444 inhabitants, and is divided into nine administrative districts [18]. The Human Development Index (HDI) of Belo Horizonte is 0.880 (HDI for income: 0.830; HDI for longevity: 0.824; HDI for education: 0.986) [19].

The sample size was calculated to give a standard error of 3.0% and a 99.0% confidence interval (CI). Based on a previous study, the prevalence of TDI was considered to be 17.1% [17]. The sample size necessary to satisfy these requirements was estimated to be 1,044 individuals. As a multistage sampling method was adopted rather than random sampling, a correction factor of 1.4 was applied to increase the precision [20], achieving a minimal sample size of 1,462. Moreover, an additional 10.0% of schoolchildren were asked to participate (n = 1,608) to compensate for potential refusals.

To ensure the representativeness of schoolchildren between 11 and 14 years of age in the city of Belo Horizonte, the Minas Gerais Board of Education was contacted to provide information on each school. The sample of schoolchildren was selected in three stages: (1) a sample was randomly selected respecting the distribution of the total number of schoolchildren in each administrative district of Belo Horizonte; (2) the number of schoolchildren in public and private schools within each administrative district was then considered for the calculation of a representative sample (Table 1); and (3) classes were randomly chosen at each selected school. All schoolchildren in selected classes who were in the range age of the study were invited to participate. Sampling was completed when the target number was reached.

**Table 1:** Frequency distribution of sample recruited (n = 1,608) by administrative district and type of school; Belo Horizonte, Brazil, 2009.

Administrative district	First stage (distribution by district)		Second stage (distribution by type of school)		
	Total of schoolchildren n (%)	Samplen (%)	Type of school	Total of schoolchildrenn (%)	Samplen (%)
Barreiro	22,129 (13.0)	209 (13.0)	Public	20,349 (92.0)	192 (91.9)
			Private	1,780 (8.0)	17 (8.1)
South Central	22,946 (13.4)	216 (13.4)	Public	13,054 (57.0)	124 (57.4)
			Private	9,892 (43.0)	92 (42.6)
East	19,972 (11.7)	188 (11.7)	Public	16,243 (81.0)	152 (80.9)
			Private	3,729 (19.0)	36 (19.1)
Northeast	20,991 (12.3)	198 (12.3)	Public	18,410 (88.0)	173 (87.4)
			Private	2,581 (12.0)	25 (12.6)
Northwest	18,988 (11.1)	179 (11.1)	Public	14,184 (75.0)	134 (74.9)
			Private	4,804 (25.0)	45 (25.1)
North	13,692 (8.1)	130 (8.1)	Public	12,635 (92.0)	119 (91.5)
			Private	1,057 (8.0)	11 (8.5)
West	16,330 (9.6)	154 (9.6)	Public	13,140 (80.0)	123 (79.9)
			Private	3,190 (20.0)	31 (20.1)
Pampulha	13,441 (7.9)	127 (7.9)	Public	9,608 (71.0)	90 (70.9)
			Private	3,833 (29.0)	37 (29.1)
Venda Nova	21,899 (12.9)	207 (12.9)	Public	20,472 (93.0)	192 (92.8)
			Private	1,427 (7.0)	15 (7.2)
Total	170,388 (100.0)	1,608 (100.0)		170,388 (100.0)	1,608 (100.0)

### 2.2. Pilot Study

A pilot study was carried out at a public school in the south-central administrative district prior to the data collection process and involved 76 schoolchildren between 11 to 14 years of age (mean age: 11.8; SD: 0.95) who did not participate in the main study. The aim of the pilot study was to test the methodology of the study (dental examinations and interviews). The results of the pilot study indicated no need to change the proposed methods. The prevalence of TDI in the pilot study was 15.8% (n = 12).

### 2.3. Clinical Oral Examination

The schoolchildren were examined by three calibrated pediatric dentists who had participated in a training and calibration exercise for the diagnosis of TDI based on the criteria proposed by Andreasen [21]. Upper and lower incisors were examined for evidence of TDI. The diagnostic criteria were non-complicated fracture (enamel and enamel-dentin fracture), complicated fracture (enamel-dentin fracture with pulp involvement), tooth dislocation (lateral luxation, intrusion and extrusion) and avulsion. It was not possible to record root fractures, as X-rays were not employed in the present study. 

The calibration exercise consisted of theoretical and clinical steps. The theoretical step involved a discussion of the Andreasen criteria for the diagnosis of TDI and an analysis of 58 photographs of TDI. A pediatric dentist, expert in TDI (the gold standard in the theoretical framework), coordinated this step. The theoretical step was performed at the School of Dentistry of the *Universidade Federal de Minas Gerais*. Forty-four children (from a school that did not take part in the main study) were selected for the calibration process. The children were examined by each pediatric dentist separately for the calculation of inter-examiner agreement and 10 were reexamined after a one-month interval for the calculation of intra-examiner agreement [17,27]. Kappa values were 1.00 for inter-examiner agreement and from 0.70 to 1.00 for intra-examiner agreement, thereby demonstrating satisfactory to excellent agreement regarding the diagnosis of TDI. 

The examinations took place at the schools during school hours. Each child was examined individually in a sitting position. Artificial illumination (Petzl Zoom head lamp, Petzl America, Clearfield, UT, USA) and disposable mouth mirrors (PRISMA®, São Paulo, Brazil) were used. The examiners used individual protection equipment. Children diagnosed with untreated TDI were interviewed to determine the history of the injury. 

### 2.4. Socioeconomic Classification

The Social Vulnerability Index (SVI) was used for the socioeconomic classification. The SVI is an area-based measure drafted for the city of Belo Horizonte and was used to analyze family exposure to social influence factors. According to the theoretical framework that supported the development of this index, social vulnerability measures the vulnerability of the population to social exclusion through the determination of neighborhood infrastructure, access to work, income, sanitation services, healthcare services, education, legal assistance and public transportation. Thus, the SVI measures social access and determines to what extent the population of each region of the city is vulnerable to social exclusion. The index is made up of five dimensions: environmental, cultural, economic, legal and security/survival. The scores for the overall SVI and each dimension of the index were calculated for each district of Belo Horizonte in a previous study [22]. For the overall SVI, higher scores denote worse conditions and greater social vulnerability. However, the scores for each dimension function in the opposite fashion, with higher scores denoting better conditions and lesser social vulnerability [17,22]. As children usually live near their schools and study in a social environment similar to that of their homes, school districts were used for this classification [17,23].

### 2.5. Statistical Analysis

Statistical analyses were performed using the Statistical Package for the Social Sciences (SPSS for Windows, version 19.0, SPSS Inc., Chicago, IL, USA). Data analysis involved descriptive statistics, such as frequency distribution, mean and standard deviation (SD). The Kolmogorov-Smirnov test demonstrated that the data obeyed non-normal distribution. Thus, the Mann-Whitney test was used to determine the statistical significance of associations between the outcome variables (TDI and seeking a dentist following TDI) and the continuous independent variables (overall SVI and its dimensions). The Chi-square test was used to determine the associations between the outcome variables and the dichotomous individual variables (gender and overjet). Principal component analysis was performed to investigate whether the five dimensions of the SVI measure a unidimensional construct. The Poisson regression model with robust variance was used for the multivariate analysis. The significance level was set at 5%.

## 3. Results

A total of 1,556 schoolchildren participated in the present study, representing 11-to-14-year-old schoolchildren of the city of Belo Horizonte, Brazil. The sample comprised 907 (58.3%) females and 649 (41.7%) males. Mean age of the participants was 12.4 (SD: 1.11). The response rate was 96.8%. The main reasons for non-response were a lack of parental agreement for the child’s participation and the child’s absence from school during the dental examination visits. As an additional 10.0% of schoolchildren were invited to participate in the study to compensate for refusals, the final sample size was slightly larger than the estimated minimal size needed to satisfy the requirements (n = 1,462).

Table 2 displays the descriptive analysis of the sample according to clinical and social vulnerability data. The prevalence of TDI was 14.1%. The majority of TDIs were fractures involving only the enamel (11.1%) and fractures involving the enamel and dentin (2.8%). As some schoolchildren had more than one type of TDI in different teeth, the sum of all types of TDI was 223. The majority of participants with TDI did not seek a dentist following the incident (58.0%). 

**Table 2:** Frequency distribution of sample (n = 1,556) by variables; Belo Horizonte, Brazil, 2009.

Variables	Frequency, n (%)	Mean (SD)
**TDI**		-
Untreated TDI	219 (14.1)	
Enamel fracture only	173 (11.1)	
Enamel-dentin fracture	43 (2.8)	
Complicated fracture	4 (0.3)	
Lateral luxation	1 (0.1)	
Avulsion	2 (0.1)	
No evidence of TDI	1,337 (85.9)	
**Etiology of TDI**		-
Falls	84 (38.3)	
Sports	42 (19.2)	
Others	26 (11.9)	
Unknown	67 (30.6)	
**Site of TDI**		-
Home	91 (41.6)	
School	23 (10.5)	
Street	26 (11.8)	
Others	14 (6.4)	
Unknown	65 (29.7)	
**Intercation with dentist following TDI **		-
Yes	57 (26.0)	
No	127 (58.0)	
Unknown	35 (16.0)	
**Overall SVI**	-	0.50 (0.16)
**Environmental dimension**	-	56.58 (18.38)
**Cultural dimension**	-	37.45 (17.16)
**Economic dimension**	-	37.89 (17.92)
**Legal dimension**	-	57.68 (20.03)
**Security/survival dimension**	-	63.67 (15.70)

Table 3 displays the associations between TDI and individual independent variables. The prevalence of TDI was higher among boys than girls (PR: 1.44; 95%CI: 1.12–1.83). The prevalence of TDI was also higher among participants with overjet greater than or equal to 5 mm in comparison to those with overjet lesser than 5 mm (PR: 1.64; 95%CI: 1.06–2.54). 

**Table 3:** Association between TDI (n = 1,556) and individual independent variables; Belo Horizonte, Brazil, 2009.

Variables	TDI		Unadjusted PR (95% CI)	*p-*value
	No evidence of TDI	Untreated TDI		
	N (%)	N (%)		
**Gender**				**0.004**
Male	538 (82.9)	111 (17.1)	1.44 (1.12–1.83)	
Female	799 (88.1)	108 (11.9)	1.00	
**Overjet**				**0.033**
≥5 mm	59 (77.6)	17 (22.4)	1.64 (1.06–2.54)	
<5 mm	1,278 (86.4)	202 (13.6)	1.00	

Chi-square test; results in bold type significant at 5% level; TDI: traumatic dental injury; PR: prevalence ratio; CI: confidence interval.

Table 4 and Table 5 display the results of the analyses of the independent variables regarding social vulnerability. Schoolchildren with untreated TDI belonged to districts with a higher overall SVI (indicating greater social vulnerability) than those without TDI (*p* = 0.036). Regarding the dimensions of SVI, poorer environmental (*p* = 0.044), economic (*p* = 0.023) and legal (*p* = 0.011) conditions were statistically associated with the presence of untreated TDI (Table 4). The children who sought dental treatment after the occurrence of TDI had lesser overall SVI scores (indicating better social status) (*p* < 0.001). These same children had higher scores for all five SVI dimensions (environmental, cultural, economic, legal and security/survival) (*p* < 0.006), indicating lesser social vulnerability. The sample size in this analysis was smaller, as it only included those children who had suffered TDI (n = 219). Thirty-five schoolchildren reported not remembering if they went to dentist or not after the incident and were excluded from this analysis. Thus, the final sample size for this analysis was 184 schoolchildren (Table 5).

Table 6 displays the loadings generated by principal component analysis, demonstrating that the five SVI dimensions measured a unidimensional construct (single component). This indicates that the five dimensions of social vulnerability measure the same thing. In the analysis, overall SVI accounted for 83.4% of the variance in the set of the five dimensions. Therefore, it was decided to use only this component (overall SVI) in the Poisson regression analysis. 

Two individual variables (gender and overjet) and one social vulnerability variable (overall SVI) were incorporated into the Poisson regression model with robust variance. The multivariate model demonstrated that the male gender (PR: 1.42; 95%CI: 1.11–1.81) and participants belonging to areas of greater social vulnerability (PR: 2.27; 95%CI: 1.11–4.61) had higher prevalence rates of untreated TDI than their counterparts. The SVI coefficient indicated an increase of 0.82 in the prevalence of TDI for each unit increase in SVI (Table 7).

**Table 4:** Association between TDI (n = 1,556) and independent variables regarding social vulnerability; Belo Horizonte, Brazil, 2009.

Subscales	TDI								*p*-value
	Untreated TDI				No evidence of TDI				
	Mean	SD	Minimum	Maximum	Mean	SD	Minimum	Maximum	
**Overall SVI**	0.52	0.15	0.12	0.79	0.49	0.16	0.12	0.79	**0.036**
**Environmental dimension**	54.62	17.77	17.27	87.17	56.91	18.46	17.27	87.17	**0.044**
**Cultural dimension**	35.36	16.28	8.95	79.50	37.79	17.28	8.95	79.50	0.082
**Economic dimension**	34.75	15.98	19.48	90.06	38.40	18.17	19.48	90.06	**0.023**
**Legal dimension**	54.69	20.28	19.97	100.00	58.17	19.94	19.97	100.00	**0.011**
**Security/survival dimension**	62.85	14.26	19.12	96.52	63.80	15.92	19.12	96.52	0.187

Mann-Whitney test; results in bold type significant at 5% level; TDI: traumatic dental injury; SVI: Social Vulnerability Index; SD: standard deviation.

**Table 5:** Association between search for dentist following TDI (n = 184) and independent variables regarding social vulnerability; Belo Horizonte, Brazil, 2009.

Subscales	Interaction with dentist following TDI								p-value
	Yes				No				
	Mean	SD	Minimum	Maximum	Mean	SD	Minimum	Maximum	
**Overall SVI**	0.47	0.17	0.12	0.79	0.55	0.13	0.16	0.79	**<0.001**
**Environmental dimension **	58.51	19.45	17.27	87.17	51.87	16.50	17.27	84.52	**<0.001**
**Cultural dimension **	40.39	17.63	8.95	79.50	32.66	15.63	8.95	79.50	**0.002**
**Economic dimension**	40.59	19.38	19.48	90.06	30.79	13.64	19.48	83.93	**<0.001**
**Legal dimension **	61.34	19.06	19.97	100.00	49.72	21.38	19.97	94.97	**<0.001**
**Security/survival dimension**	64.45	17.61	19.12	96.52	61.78	12.04	19.12	84.31	**0.006**

Mann-Whitney test; results in bold type significant at 5% level; TDI: traumatic dental injury; SVI: Social Vulnerability Index; SD: standard deviation.

**Table 6:** Principal component analysis (Varimax), loadings and single component generated by five dimensions of social vulnerability.

Dimensions	Component label
	1 “Social vulnerability”
**Environmental dimension**	0.951
**Cultural dimension**	0.956
**Economic dimension**	0.927
**Legal dimension**	0.865
**Security/survival dimension**	0.866

**Table 7:** Poisson regression model explaining individual and neighborhood variables in schoolchildren with untreated TDI (1,556); Belo Horizonte, Brazil, 2009.

Variables	Coefficient (95% CI)	Adjusted PR (95% CI)	Hypothesis Test	
			Chi-square	*p*-value
**Gender**	0.35 (0.10–0.60)		7.76	**0.005**
Male		1.42 (1.11–1.81)		
Female		1.00		
**Overjet**	0.43 (−0.01–0.87)		3.69	0.055
≥5 mm		1.54 (0.99–2.38)		
<5 mm		1.00		
**SVI**	0.82 (0.11–1.53)	2.27 (1.11–4.61)	5.07	**0.024**

Poisson regression model with robust variance; results in bold type significant at 5% level; PR: prevalence ratio; CI: confidence interval; SVI: Social vulnerability index.

## 4. Discussion

The present study demonstrated that the occurrence of TDI and subsequent search for treatment were associated with social vulnerability. The prevalence of TDI was greater among schoolchildren from regions of high social vulnerability and a lower frequency of seeking a dentist for restorative treatment following a TDI was reported by schoolchildren from such regions. These findings offer an additional knowledge on the influence of socioeconomic factors with regard to the occurrence of TDI. The local index (SVI) employed in the present study measures five dimensions of citizenship composed of eight indicators. SVI is the result of the mapping of various manifestations in Belo Horizonte using geo-referenced information. This mapping resulted in the division of the city into 81 planning units. Each planning unit received a score based on the indicators that showed how much the population of each unit was excluded from access to certain dimensions of citizenship and related these results with demographic characteristics, extremes of exclusion or inclusion and issues related to protective social services [22]. 

The large sample size and the employment of an index that measures the dimensions of citizenship make a unique contribution to the literature on the association between socioeconomic conditions and the occurrence of TDI. The SVI is a local index that permits identifying regions of the city in which the population is more vulnerable to social exclusion as well as the aspects to which the population is more vulnerable [22]. The SVI provides more precise knowledge on social disparities within the city and can help guide decisions regarding adequate public policies [22].

The findings of the present study support the hypothesis that children who live and study in areas of greater social vulnerability are more likely to exhibit untreated TDI than those from areas of lesser social vulnerability. This result is true for the overall SVI as well as for the environmental, economic and legal dimensions of citizenship. It has been reported that current vulnerability status affects a child’s health status by influencing parents’ ability to offer adequate care [24]. These findings are in agreement with precepts put forth by the World Health Organization [25] stating that the prevention and control of chronic diseases require a global strategy based on interventions directed at environmental, economic, social and behavioral determinants. 

In the present study, the environmental dimension was assessed by indicators of housing quality, household density and available urban infrastructure [22]. The results corroborate those reported in previous studies involving Brazilian preschool children as well as schoolchildren from Brazil, Thailand and Canada, which demonstrate the importance of the neighborhood and school environments with regard to the occurrence of TDI [1,2,3,4,14,26]. Children enrolled in more supportive schools that include health promotion activities are more likely to exhibit better oral health than those in non-supportive schools [4,26]. The community in which an individual lives may play an important role in the establishment of oral health. One theoretical model, denominated the “salutogenic model”, supports the hypothesis that all individuals are exposed to stressors and the manner by which each one copes with these stressors is strongly associated with a sense of coherence on both the individual and community levels [27]. Variables such as adequate housing conditions and safe working conditions are examples of salutary factors that have a positive influence on oral health as well as other health outcomes [27].

The economic dimension was also associated with untreated TDI in the present study. This finding was expected, as a number of previous studies report such an association [9,10,11]. Two of the studies cited measured economic status based on parents’ employment, education level and family income [10,11]. The other study employed the criteria of the Brazilian Advertising Association/Brazilian Association of Market Institutes, which classify the population in socioeconomic classes by estimating buying power [9]. The criteria used in the present study (economic dimension of the SVI) were based on employment status and income [22]. Although previous studies have not found an association between socioeconomic status and TDI [7,8,12,13], it is important to note that this variable was strongly association with the presence of untreated TDI and the search for dental treatment following the incident. In the present study, schoolchildren who sought dental care following a TDI belonged to areas of lesser social vulnerability than those who did not seek treatment.

The search for dental care after the occurrence of TDI was significantly associated with the overall SVI and all five dimensions of citizenship. These findings demonstrate the difficulty in achieving access to dental care in a developing country, such as Brazil, in which the majority of the population cannot afford private dental care and public services are unable to offer complex treatment [17]. Thus, the results of the present study confirm the hypothesis that children from areas of lesser social vulnerability have greater access to dental care concerning all aspects analyzed (environmental, cultural, economic, legal and security/survival). The difference in the cultural dimension could be explained by the fact that TDI is not considered a disease by most parents and those parents with lower access to education do not pay it concern [17,28]. Previous studies report that restorative treatment for a TDI has been neglected [16,17,28], although the literature has demonstrated that severe TDI may have physical and psychosocial consequences [11,29,30]. The studies cited also found that treatment often does not eliminate the negative impact of TDI, but can reduce this impact, especially with regard to psychosocial aspects [11,30].

The present study has limitations that must be recognized. The clinical examinations were performed in the schools, which did not allow the use of X-rays. Thus, diagnoses were obtained through a visual dental examination alone, which may have led to the underestimation of TDI due to the inability to detect root fractures. However, this diagnostic procedure allowed obtaining a large population-based sample size with an epidemiological nature representative of the city of Belo Horizonte (Brazil). A non-respondent selection bias may have occurred due to the refusal of some schoolchildren to participate in the study. This kind of bias occurs when there is a strong difference between respondents and non-respondents [31]. However, the non-response rate in the present study was very small, as only 3.2% of the recruited schoolchildren refused to participate. Moreover, a correction factor of 1.4 was applied to the calculation of the sample size to increase the statistical precision and minimize this potential problem.

For many years, the approach to health has been focused on prevention, in which changes in life style and behavior are the consequence of education. However, the prevention model has limitations with regard to improving health [24,27]. Recent studies have demonstrated the value of health promotion policies directed at health determinants [3,4]. This new approach is based on detecting inequalities and recognizing social and environmental conditions. The level of social trust, collective benefits and the degree of involvement in social and community issues are important factors to establishing behavior that favors oral health [3,4,27]. Epidemiology should move towards the involvement of global health issues, with researchers and governments working together to improve the overall health of the population [32] by promoting environmental actions aimed at facilitating healthier choices [27].

## 5. Conclusions

In summary, the results of the present study support the hypothesis that the male gender and individuals belonging to areas of greater social vulnerability are more likely to experience TDI than their counterparts. Greater social vulnerability is also associated with a lesser frequency of seeking dental treatment following the occurrence of TDI. The present study demonstrates the importance of using local indices comprising the characteristic peculiarities of each particular site in order to measure socioeconomic conditions. This study also demonstrates the importance of addressing different dimensions of citizenship to gain a better understanding of the association between socioeconomic status and oral health.
